# Impact of introduction of rapid diagnostic tests for malaria on antibiotic prescribing: analysis of observational and randomised studies in public and private healthcare settings

**DOI:** 10.1136/bmj.j1054

**Published:** 2017-03-29

**Authors:** Heidi Hopkins, Katia J Bruxvoort, Matthew E Cairns, Clare I R Chandler, Baptiste Leurent, Evelyn K Ansah, Frank Baiden, Kimberly A Baltzell, Anders Björkman, Helen E D Burchett, Siân E Clarke, Deborah D DiLiberto, Kristina Elfving, Catherine Goodman, Kristian S Hansen, S Patrick Kachur, Sham Lal, David G Lalloo, Toby Leslie, Pascal Magnussen, Lindsay Mangham Jefferies, Andreas Mårtensson, Ismail Mayan, Anthony K Mbonye, Mwinyi I Msellem, Obinna E Onwujekwe, Seth Owusu-Agyei, Hugh Reyburn, Mark W Rowland, Delér Shakely, Lasse S Vestergaard, Jayne Webster, Virginia L Wiseman, Shunmay Yeung, David Schellenberg, Sarah G Staedke, Christopher J M Whitty

**Affiliations:** 1 London School of Hygiene and Tropical Medicine, London WC1E 7HT, UK; 2 Ghana Health Service, Accra, Ghana; 3 Ensign College of Public Health, Kpong, Ghana; 4 University of California, San Francisco, CA, USA; 5 Karolinska Institutet, Stockholm, 17176, Sweden; 6 University of Gothenburg, Gothenburg, Sweden; 7 University of Copenhagen, Copenhagen, DK1014, Denmark; 8 US Centers for Disease Control and Prevention, Atlanta, GA, USA; 9 Liverpool School of Tropical Medicine, Liverpool, UK; 10 Health Protection Research Organisation, Kabul, Afghanistan; 11 Centre for Medical Parasitology, University of Copenhagen and Copenhagen University Hospital, and Department for Veterinary Disease Biology, University of Copenhagen, Copenhagen, Denmark; 12 Uppsala University, Uppsala, Sweden; 13 Ministry of Health, Kampala, Uganda; 14 Makerere University School of Public Health, Kampala, Uganda; 15 Zanzibar Malaria Elimination Programme, Tanzania; 16 Department of Pharmacology and Therapeutics, University of Nigeria, Enugu, Nigeria; 17 Kintampo Health Research Centre, Kintampo, Ghana; 18 Centre for Malaria Research, Karolinska Institutet, Stockholm, Sweden, and Health Metrics at Sahlgrenska Academy, University of Gothenburg, Gothenburg, Sweden; 19 Department of Infectious Disease Epidemiology, Statens Serum Institut, Copenhagen, Denmark; 20 School of Public Health and Community Medicine, University of New South Wales, Sydney, Australia

## Abstract

**Objectives** To examine the impact of use of rapid diagnostic tests for malaria on prescribing of antimicrobials, specifically antibiotics, for acute febrile illness in Africa and Asia.

**Design** Analysis****of nine preselected linked and codesigned observational and randomised studies (eight cluster or individually randomised trials and one observational study).

**Setting** Public and private healthcare settings, 2007-13, in Afghanistan, Cameroon, Ghana, Nigeria, Tanzania, and Uganda.

**Participants** 522 480 children and adults with acute febrile illness.

**Interventions** Rapid diagnostic tests for malaria.

**Main outcome measures** Proportions of patients for whom an antibiotic was prescribed in trial groups who had undergone rapid diagnostic testing compared with controls and in patients with negative test results compared with patients with positive results. A secondary aim compared classes of antibiotics prescribed in different settings.

**Results** Antibiotics were prescribed to 127 052/238 797 (53%) patients in control groups and 167 714/283 683 (59%) patients in intervention groups. Antibiotics were prescribed to 40% (35 505/89 719) of patients with a positive test result for malaria and to 69% (39 400/57 080) of those with a negative result. All but one study showed a trend toward more antibiotic prescribing in groups who underwent rapid diagnostic tests. Random effects meta-analysis of the trials showed that the overall risk of antibiotic prescription was 21% higher (95% confidence interval 7% to 36%) in intervention settings. In most intervention settings, patients with negative test results received more antibiotic prescriptions than patients with positive results for all the most commonly used classes: penicillins, trimethoprim-sulfamethoxazole (one exception), tetracyclines, and metronidazole.

**Conclusions** Introduction of rapid diagnostic tests for malaria to reduce unnecessary use of antimalarials—a beneficial public health outcome—could drive up untargeted use of antibiotics. That 69% of patients were prescribed antibiotics when test results were negative probably represents overprescription.****This included antibiotics from several classes, including those like metronidazole that are seldom appropriate for febrile illness, across varied clinical, health system, and epidemiological settings. It is often assumed that better disease specific diagnostics will reduce antimicrobial overuse, but they might simply shift it from one antimicrobial class to another. Current global implementation of malaria testing might increase untargeted antibiotic use and must be examined.

## Introduction

There is wide recognition that overuse of antimicrobials drives resistance in micro-organisms.^1^ ^2^ ^3^ ^4^ Global concern is growing in the face of accumulating evidence showing international and intercontinental spread of bacterial resistance.^2^ ^3^ ^5^ Dealing this crisis has become a major priority, with the World Health Assembly adopting a global action plan in 2015.^6^ Several strategies have been proposed to contain the risks of antimicrobial resistance, including improved surveillance, optimised use of available antibiotics, development of new antibiotics, and development of better diagnostic tests. Tackling antimicrobial resistance will require sustained cooperation across international borders and agencies.^7^ ^8^

Across tropical and subtropical zones in Africa and Asia, acute febrile illness is one of the most common reasons for people to seek treatment from health services.^9^ Historically, many if not most fevers have been considered to result from malaria and have been treated empirically with antimalarials.^10^ Many other infectious and non-infectious diseases, however, can cause similar symptoms, including bacterial and viral infections. Management of fever has received considerable attention in recent years, with widespread efforts, spearheaded by WHO, to improve diagnosis of malaria and reduce untargeted use of antimalarials.^11^ ^12^ These efforts have relied heavily on introduction of rapid diagnostic tests for malaria. These tests detect parasite antigen in a fingerprick blood sample and are seen as simple and quick to use compared with the traditional diagnostic method of microscopy. Each year, millions of rapid diagnostic tests are now used in diverse healthcare settings across endemic areas.^13^ In many settings this has led to a reduction in overprescription of antimalarials, but the impact on use of other treatments is less clear.^14^ There are now calls to expand disease specific rapid diagnostics as a solution to other antimicrobial prescribing challenges.^15^

We hypothesised that improved malaria diagnosis to reduce use of antimalarials, a widely used antimicrobial class, could paradoxically drive an increase in untargeted use of other antimicrobials, such as antibiotics, particularly when test results for malaria are negative.^16^ Little is known about the causes of non-malaria febrile illness in many malaria endemic countries,^17^ where microbiological diagnostic facilities are limited or non-existent in most settings. Health workers can resort only to educated guesswork and empirical treatment for febrile patients who do not have malaria. Improvements in malaria diagnosis could simply reduce the overuse of antimalarials, or divert overuse to other products like antipyretics, or it could divert antimalarial overuse to other antimicrobials, particularly antibiotics.^18^ Prescribing practices are not well documented or regulated in regions with little healthcare infrastructure and with relatively unrestricted access to antimicrobials.^18^ ^19^ ^20^ ^21^ ^22^ ^23^ ^24^ Similarly, monitoring and surveillance of antimicrobial resistance is not conducted in most lower and middle income countries, but available data do show clinically relevant resistance in many common bacterial pathogens.^25^ ^26^ ^27^ ^28^ ^29^ ^30^ ^31^ ^32^ ^33^ ^34^ ^35^

The ACT Consortium (www.actconsortium.org) included several studies that evaluated the potential of rapid diagnostic tests for malaria to improve case management for patients with undifferentiated fever in malaria endemic areas. Data from these studies, conducted in multiple geographical, epidemiological, and health system settings, provide the largest and most varied sample to date to assess whether changes in antimalarial prescribing behaviour are associated with shifts in antibiotic prescribing. To inform policy for treatment of febrile illness, we compared settings where tests were and were not implemented, examined the differences in antibiotic prescribing overall and by test result, and identified the antibiotic classes used in different settings.

## Methods

### Overview of studies included in analysis

The ACT Consortium conducted linked and co-designed research studies in Africa and Asia, including multiple studies designed to evaluate the impacts on healthcare of introducing rapid diagnostic tests for malaria in various settings. The studies were designed to be complementary and mutually reinforcing and to cover a range of settings. These rapid diagnostic tests were introduced in various epidemiological settings and health service sectors (public, private retail, and community) and with different approaches to implementation. To avoid selection bias, our combined analysis includes all studies in the ACT Consortium that were designed a priori to test the effect of introduction of rapid diagnostic tests for malaria on prescribing of antimalarial drugs, where providers could prescribe antibiotics, and did not include any other studies post hoc. Detailed descriptions of the individual studies are available in open access publications.^36^ ^37^ ^38^ ^39^ ^40^ ^41^ ^42^ ^43^****

We included in the analysis studies that met the following criteria: evaluated an intervention to implement rapid diagnostic tests for malaria in settings where participating providers could prescribe both antimalarials and antibiotics, compared sites with and without the intervention, documented prescriber behaviour as a primary outcome, and collected individual patient data on diagnostic test results and treatments prescribed including antibiotics. Tables 1 and 2[Table tbl1 tbl2] present descriptions of the nine studies meeting these criteria. In all of the studies the prescribers used rapid diagnostic tests for illness syndromes that clinically could have been malaria.

**Table 1 tbl1:** Description of study contexts and populations of patients according to whether they underwent rapid diagnostic test for malaria (mRDT)

Region and study abbreviation	No of patient encounters		Percentage (No) of positive test results in symptomatic patients^*^	Setting	Healthcare sector	Median (IQR) patient age (years)	Study dates
	Control	mRDT					
East Afghanistan (Afgh-com/a)	607	733	28.8 (208/723)	Urban and rural	Community	14 (8-30)	Oct 2011-May 2012
North Afghanistan (Afgh-com/b)	594	466	(1/463)				
East Afghanistan (Afgh-pub/a)	2005	2048	27.1 (555/2048)	Urban and rural	Public	13 (7-25)	Sept 2009-Sept 2010
North Afghanistan (Afgh-pub/b)	840	856	(7/856)				
West Cameroon (Cam-pub/a)	400	1477	18.4 (202/1098)	Urban and rural	Public/mission	13 (3-29)	Oct-Dec 2011
Central Cameroon (Cam-pub/b)	281	1824	50.6 (715/1412)				
South east Ghana (Ghan-pub)	3634	3629	36.0 (1308/3629)	Rural	Public	13 (4-32)	Aug 2007-Dec 2008
North Tanzania (Tanz1-pub/a)	689	750	21.4 (77/360)	Rural/peri-urban	Public	2 (1-17)	May-Oct 2010; April-July 2012^†^
West Tanzania (Tanz1-pub/b)	559	388	10.8 (18/167)				
South east Tanzania (Tanz1-pub/c)	498	572	46.9 (192/409)				
North east Tanzania (Tanz2-pub)	16 068	44 121	25.4 (4400/17 297)	Rural	Public	11 (2-31)	Sept 2010-Jan 2011; Feb 2011-March 2012^†^
South east Uganda (Uga-pub)	210 758	221 755	69.5 (81 359/117 070)	Rural	Public	12 (3-28)	April 2011-March 2013
South central Uganda (Uga-priv)	8109^‡^	10 365^‡^	57.0 (5690/9987)	Rural	Private retail	8 (2 – 22)	Jan–Dec 2011
South central Nigeria (Nige-mix)	1642	4946	52.3 (589/1126)	Urban and rural	Public and private retail	26 (18 – 35)	July–Dec 2009; June-Dec 2011^†^

IQR=interquartile range.

*Proportion of patients testing positive for malaria (among those in intervention settings who were tested) presented as proxy for malaria epidemiology.

†Ranges separated by semicolon indicate pre-/post- evaluations conducted before and after introduction of mRDTs. Other studies consisted of multiple study arms without (control) and with (intervention) mRDTs, evaluated simultaneously.

‡In Uga-priv only a subset of patients (n=497; 248 in control setting, 249 in intervention setting) were followed up after consultation to collect data on medicines prescribed.

**Table 2 tbl2:** Description of study designs and interventions in patients who underwent rapid diagnostic test for malaria (mRDT)

Study abbreviation	Design	Tests used^*^	Training^†^ provided with test introduction in intervention settings
Afgh-com	Cluster randomised trial	CareStart Pf/Pan, AccessBio	One day MoH training: performing mRDTs and prescribing antimalarials
Afgh-pub	Individually randomised trial	CareStart Pf/Pan, AccessBio	One day MoH training: performing mRDTs and prescribing antimalarials
Cam-pub	Cluster randomised trial	SD Bioline Malaria Ag Pf/Pan, Standard Diagnostics	One day training: performing mRDTs, prescribing antimalarials. Enhanced training group: additional two day interactive training on adapting to the malaria guideline change including identifying major alternative causes of febrile illness, and communication skills
Ghan-pub	Individually randomised trial	OptiMAL-IT, Diamed AG	Two day training: use of mRDTs, antimalarial prescribing, identifying major alternative causes of febrile illness
Tanz1-pub	Baseline and follow-up surveys before and after mRDT introduction	Primarily SD Bioline Pf, Standard Diagnostics	Two day MoH training: performing mRDTs, prescribing antimalarials, rationale for malaria guideline change, identifying major alternative causes of febrile illness
Tanz2-pub	Baseline survey followed by cluster randomised trial	Paracheck Pf, Orchid Biomedical Systems	Two day MoH training: performing mRDTs, prescribing antimalarials, rationale for malaria guideline change, identifying major alternative causes of febrile illness. Enhanced training group: three additional half day workshops on adapting to and sustaining guideline change, and communication skills
Uga-pub	Cluster randomised trial	Primarily SD Bioline Pf, or SD Bioline Pf/Pan, Standard Diagnostics	Two day training plus on site supervision: performing mRDTs, prescribing antimalarials, identifying major alternative causes of febrile illness, and communication skills
Uga-priv	Cluster randomised trial	First Response Ag Pf card, Premier Medical Corporation	Four day training: performing mRDTs, prescribing antimalarials, referral algorithm for mRDT-negative results, and communication skills
Nige-mix	Formative study followed by cluster randomised trial	SD Bioline Malaria Ag Pf/Pan, Standard Diagnostics	Half day training: demonstration on mRDT use. Enhanced training group: additional two day training on performing mRDTs, prescribing antimalarials, and communication skills

MoH=Ministry of Health.

*mRDTs selected in agreement with national health authorities and supplied by studies in most cases, except in Tanz1-pub where they were supplied by MoH as part of national scale-up, and in Uga-pub where they were supplied by MoH with study back-up in case of stockouts of antibiotics because of intermittent supply.

†Many training programmes included information on identifying alternative causes of fever; none included systematic guidance on management of alternative causes of fever.

The studies were conducted in 2007-13 in Afghanistan, Cameroon, Ghana, Nigeria, Tanzania, and Uganda in a mix of rural and urban settings. Rapid diagnostic tests were introduced among government sponsored community health workers (Afgh-com (T Leslie, et al, in preparation)), in public health facilities only (Afgh-pub,^36^ Cam-pub,^37^ Ghan-pub,^38^ Tanz1-pub,^40^ Tanz2-pub,^39^ Uga-pub^43^), in private drug shops only (Uga-priv^41^), and in a combination of public facilities, private pharmacies, and drug shops (Nige-mix^42^). Most studies included were cluster randomised trials of interventions, with the exception of two individually randomised trials (Afgh-pub,^36^ Ghan-pub^38^), and one descriptive study before and after national implementation of rapid diagnostic tests (Tanz1-pub^40^). Table 2 summarises the intervention in each study.

Microscopy services were not present or were limited in five study settings.^36^ ^39^ ^41^ ^42^ ^43^ In Cam-pub, microscopy was widely available and its use increased during the time of the trial alongside a national malaria campaign.^37^ In Tanz1-pub, microscopy was available in some higher level facilities but was not frequently used.^40^ The two individually randomised studies (Afgh-com (T Leslie, et al, in preparation), Ghan-pub^38^) introduced rapid diagnostic tests in some settings where routine care included microscopy. In other countries, the effect of introducing tests was evaluated against control settings where presumptive clinical diagnosis was the norm.

Prescribing data were collected through patient exit interviews or records of treatments administered (“registers”) completed by the provider, both of these methods, and both registers and follow-up interviews for a subset of patients (Uga-priv).^41^

The main outcome of interest was the proportion of patients in each setting who were prescribed at least one systemic (oral or injectable) antibiotic. Other outcomes included the type of antibiotic prescribed.

### Patient involvement

The development of the primary research studies, from which these data are drawn, was informed by formative research among health workers, community members, and other stakeholders in the study settings; details for individual studies are available in open access publications.^44^ ^45^ ^46^ ^47^ Results of the individual trials were disseminated to participants in their local contexts. Patients were not directly involved in the design of the present analysis.

### Analysis approach

We performed three analyses to represent different policy and clinical perspectives. The main outcome for the first two analyses is the risk ratio of being prescribed at least one systemic (oral or injectable) antibiotic. The first analysis compared overall antibiotic prescribing for each study, in settings with and without rapid diagnostic test interventions; this represents the overall policy effect of introducing diagnostic tests in a given context. The second, restricted to patients in intervention settings, looked at those with positive test result compared with those with negative results. This analysis shows the effect of test result on antibiotic prescribing. In the third analysis, we categorised prescribed antibiotics by drug class to explore the range of antibiotic classes used in different settings. We also examined the impact of rapid diagnostic tests on prescription of the most commonly used antibiotic classes, defined as those that were prescribed to at least 5% of patients in at least one site, to see if there was a differential effect of test result by antibiotic class.

To allow comparison of the impact of introduction of rapid diagnostic tests for malaria and test results across studies, we calculated the risk ratios with their 95% confidence intervals for each study using binomial regression with a log link. The Huber-White robust standard error was used to account for correlation within the highest level of clustering (that is, within randomisation clusters for the cluster randomised trials and within study clinics for the individually randomised studies).^48^ We carried out a random effects meta-analysis including all studies that compared groups randomised concurrently to intervention or control—that is, excluding Tanz-1, which was a before and after comparison, and Nige-mix and Tanz-2, which compared groups randomised to interventions with pre-intervention baseline data. For these three studies, table 3 shows estimates of the impact of test introduction, but they do not contribute to the formal meta-analysis.

**Table 3 tbl3:** Malaria diagnostic testing, test results, and antibiotic prescribing in control areas compared with areas where rapid diagnostic tests for malaria (mRDTs) were introduced

Study	Percentage (No) who underwent diagnostic test			Percentage (No) of those tested whose result was negative			Percentage (No) prescribed at least one antibiotic		Risk ratio for antibiotic prescription in mRDT area *v* control area (95% CI)
	Control^*^	mRDT		Control^*^	mRDT		Control^*^	mRDT	
Afgh-com/a	0 (0)	98.8 (724/733)		0^†^	71.2 (515/723)		18.2 (110/605)	54.1 (383/708)	2.98 (1.62 to 5.5)
Afgh-com/b	0 (0)	100 (466/466)		0^†^	99.8 (462/463)		48.4 (286/591)	68.5 (317/463)	1.41 (0.93 to 2.15)
Afgh-pub/a	100^‡^ (2005/2005)	100^‡^ (2048/2048)		67.9 (1357/2000)	72.9 (1493/2048)		38.1 (763/2005)	40.8 (836/2048)	1.07 (0.99 to 1.17)
Afgh-pub/b	55.5^‡^ (466/840)	100^‡^ (856/856)		96.6 (450/466)	99.2 (849/856)		50.6 (425/840)	70.6 (604/856)	1.39 (0.99 to 1.97)
Cam-pub/a	78.3 (313/400)	75.3 (1111/1475)		75.6 (232/307)	81.6 (896/1098)		72.8 (287/394)	78.5 (1130/1439)	1.08 (0.93 to 1.26)
Cam-pub/b	80.4 (226/281)	79.5 (1448/1822)		6.0 (13/218)	49.4 (697/1412)		50.4 (140/278)	52.1 (925/1774)	1.03 (0.66 to 1.63)
Ghan-pub	52.5^‡^ (1908/3634)	100^‡^ (3629/3629)		69.7 (1320/1907)	64.0 (2321/3629)		29.5 (1069/3623)	32.3 (1168/3615)	1.10 (0.97 to 1.24)
Tanz1-pub/a^§^	7.3 (50/689)	48.4 (363/750)		50.0 (25/50)	78.6 (283/360)		29.7 (204/688)	44.7 (335/749)	1.51 (1.12 to 2.03)
Tanz1-pub/b^§^	12.7 (71/559)	43.2 (167/387)		50.7 (36/71)	89.2 (149/167)		35.2 (197/559)	56.4 (219/388)	1.60 (1.27 to 2.02)
Tanz1-pub/c^§^	31.3 (156/498)	71.5 (409/572)		44.9 (70/156)	53.1 (217/409)		33.1 (165/498)	49.0 (280/572)	1.48 (1.19 to 1.84)
Tanz2-pub	0 (0/16 068)	39.8^¶^ (17 559/44 119)		0^†^	74.6 (12 897/17 297)		61.5 (9875/16 068)	73.2 (32 274/44 121)	1.19 (1.13 to 1.25)
Uga-pub	7.3 (15 285/210 758)	52.9 (117 350/210 578)		41.3 (6261/15 171)	30.5 (35 711/117 070)		53.7 (113 101/ 210 758)	57.9 (128 404/221 755)	1.08 (0.90 to 1.30)
Uga-priv	0 (0/8109)	97.3 (10 078/10 365)		0^†^	43.0 (4297/9987)		19.4 (48/248)^**^	34.9 (87/249)^**^	1.80 (1.30 to 2.50)
Nige-mix	1.7 (27/1634)	23.1 (1137/4922)		0 (0/25)	47.7 (537/1126)		23.3 (382/1642)	15.2 (752/4946)	0.65 (0.46 to 0.93)

*Microscopy services were not present or were limited in five study settings. In Cam-pub, microscopy was widely available and its use increased during the time of the trial alongside a national malaria campaign. In Tanz1-pub, microscopy was available in some higher level facilities but was not frequently used. The two individually randomised studies (Afgh-com, Ghan-pub) introduced rapid diagnostic tests in some settings where routine care included microscopy. In other countries, the effect of introducing tests was evaluated against control settings where presumptive clinical diagnosis was the norm.”

†No observations (no testing available or performed in control area).

‡Afgh-pub and Ghan-pub were individually randomised trials; all patients in intervention group were tested with malaria rapid diagnostic test (mRDT); in control group, half were tested by microscopy, and half were diagnosed clinically.

§Tanz1 recorded medicines actually obtained by patients; other studies recorded medicines prescribed.

¶Figure is lower than that reported in primary paper^39^ because the present analysis included all patients with data, rather than only patients defined by study as mRDT-eligible.

**Only subset of patients (n=497) in Uga-priv followed up after consultation to collect data on medicines prescribed.

## Results

Table 1 describes the study sites, including studies in Afghanistan (two), Cameroon, Ghana, Nigeria, Tanzania (two), and Uganda (two), and covering a range of epidemiological settings. Data from the nine sites in six countries represent 522 480 patients with febrile illness or other malaria-like presentations.

Of patients in intervention settings for whom a malaria test result (slide or rapid diagnostic test) was available, 61 324/157 345 (39.0%) tested negative for malaria, ranging from 30.5% (35 711/117 070 in southeast Uganda) to 99.8% (462/463 in northern Afghanistan). Parasite prevalence acts as a proxy for local endemicity.

Table 3 shows overall data on diagnostic testing and antibiotic prescribing for each study setting. In intervention settings, the proportion of patients for whom a test was performed varied from 23.1% (1137/4922) to 99.8% (724/733). The heterogeneity of uptake of rapid test results is important to the generalisability of these data to different settings.

### Antibiotic prescription in settings with and without interventions of rapid diagnostic tests for malaria

Antibiotics were prescribed to 127 052/238 797 (53%) patients in control groups and 167 714/283 683 (59%) in intervention groups. The proportion of patients prescribed at least one systemic (oral or injectable) antibiotic ranged from 18.2% (110/605) to 72.8% (287/394) in control settings and from 15.2% (752/4946) to 78.5% (1130/1439) in settings with a rapid test intervention. Relative to control settings, the proportion of patients receiving an antibiotic prescription was higher or had a trend towards being higher where rapid diagnostic tests were introduced in all but one of the studies (in Nigeria), with risk ratios ranging from 0.65 to 2.98 (fig 1, and table 3). This represents the overall impact on health systems of introducing rapid diagnostic tests. A meta-analysis combining the randomised comparisons gave an overall risk ratio of 1.21 (95% confidence interval 1.07 to 1.36; P=0.004)—that is, the risk of antibiotic prescription was 21% higher where rapid diagnostic tests were introduced, although there was an important heterogeneity between studies (I^2^=65%).

**Figure f1:**
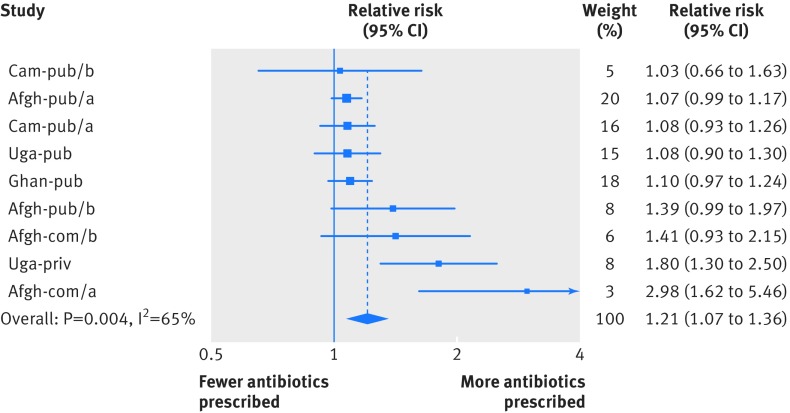
**Fig 1** Risk ratios for antibiotic prescription in randomised studies comparing patients in control settings with patients in settings where malaria rapid diagnostic test intervention was implemented. Weights are from random effects analysis

### Antibiotic prescription according to malaria test results

In intervention settings across all studies, antibiotic prescribing was higher among patients with negative malaria test results than among patients with positive results (fig 2 and table 4). Antibiotics were prescribed to 40% (35 505/89 719) of patients with a positive test result and to 69% (39 400/57 080) of those with a negative result. Differences were substantial in several studies (Afgh-com/a, Afgh-pub/a and b, Cam-pub/a and b, Ghan-pub, Tanz1-pub/a and c, Tanz2-pub, Uga-pub and Uga-priv), with risk ratios overall varying from 1.13 to 15.17. This represents the impact of health workers obtaining a test result negative for malaria.

**Figure f2:**
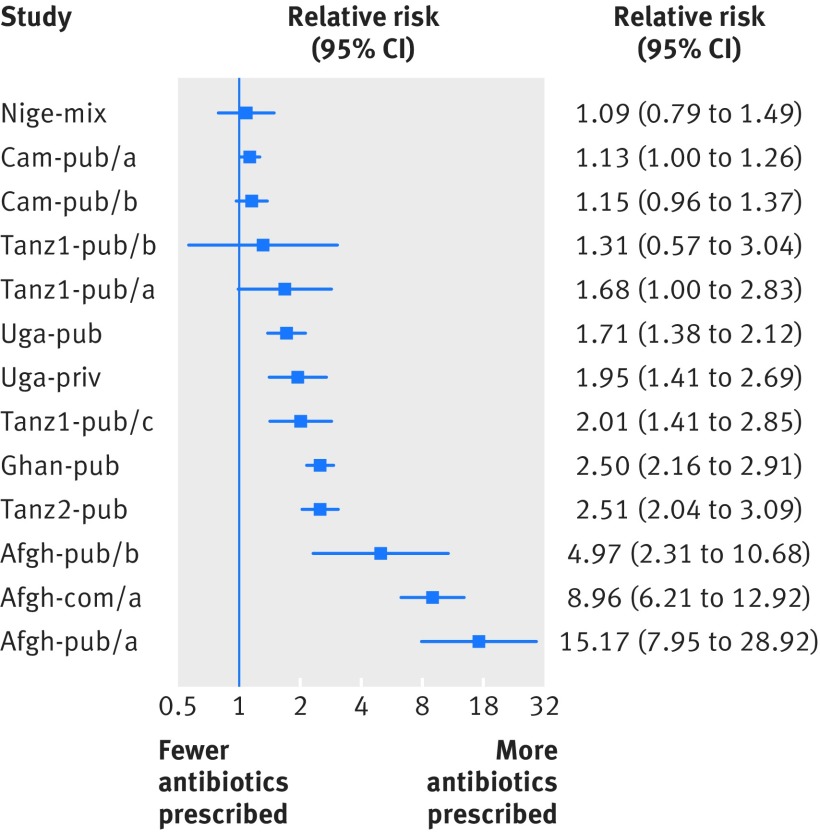
**Fig 2** Risk ratios for antibiotic prescription in settings with malaria rapid diagnostic test intervention, comparing patients with positive versus negative malaria test results. Afgh-com/b is not included because risk ratio could not be calculated; comparison is not possible when no patients with positive test results received antibiotic

**Table 4 tbl4:** Antibiotic prescribing by test result in areas with rapid diagnostic test for malaria (mRDT) intervention

Study	Percentage (No) of patients prescribed at least one antibiotic^*^		Risk ratio for antibiotic prescription for test negative *v* test positive patients (95% CI)
	Negative test result	Positive test result	
Afgh-com/a	72.8 (366/503)	8.1 (16/197)	9.0 (6.2 to 12.9)
Afgh-com/b	68.9 (316/459)	0 (0/1)	—^†^
Afgh-pub/a	54.7 (816/1493)	3.6 (20/555)	15.2 (8.0 to 28.9)
Afgh-pub/b	71.0 (603/849)	14.3 (1/7)	5.0 (2.31 to 10.7)
Cam-pub/a	81.6 (718/880)	72.5 (140/193)	1.13 (1.00 to 1.26)
Cam-pub/b	58.5 (397/679)	50.9 (356/700)	1.15 (0.96 to 1.37)
Ghan-pub	41.3 (953/2310)	16.5 (215/1305)	2.50 (2.16 to 2.91)
Tanz1-pub/a^‡^	53.0 (150/283)	31.6 (24/76)	1.68 (1.00 to 2.83)
Tanz1-pub/b^‡^	58.4 (87/149)	44.4 (8/18)	1.31 (0.57 to 3.04)
Tanz1-pub/c^‡^	61.8 (134/217)	30.7 (59/192)	2.01 (1.42 to 2.85)
Tanz2-pub	75.6 (9750/12 897)	30.1 (1326/4400)	2.51 (2.04 to 3.09)
Uga-pub	69.9 (24 963/35 711)	40.8 (33 214/81 359)	1.71 (1.38 to 2.12)
Uga-priv	46.0 (52/113)	23.6 (30/127)	1.95 (1.41 to 2.69)
Nige-mix	17.7 (95/537)	16.3 (96/589)	1.09 (0.79 to 1.49)

*Where denominators do not sum to total, uptake of mRDTs by clinicians was not 100%, so that mRDT not performed for proportion of patients seen.

†Comparison not possible where no mRDT-positive patients received antibiotic.

‡Tanz1 recorded drugs actually obtained by patients; other studies recorded medicines prescribed.

### Types of antibiotics prescribed

Table 5 shows the percentage of patients at each site who were prescribed each class of antibiotic. Figure 3 shows the percentage contribution of each antibiotic class to total antibiotic prescribing at each site. Penicillins and trimethoprim-sulfamethoxazole (TMP-SMX, or cotrimoxazole) were the most commonly prescribed antibiotics, with metronidazole the third most prescribed antibiotic at most sites. In Cam-pub, Nige-mix, and Tanz1-pub the type of antibiotic prescribed was not known in 52.1% (2075/3982), 10.7% (708/6588), and 15.9% (548/3456) of cases, respectively, because of the data collection approach or coding.

**Table 5 tbl5:** Proportion (number) of all patients seen who were prescribed each class of antibiotics^*^

	Penicillin	Cephalo-sporin	Macrolide	Tetracycline	TMP/SMX	Quinolone	Chloramphenicol	Amino-glycoside (gentamicin)	Metronidazole	Other	Type unknown
Afgh-pub^†^ (n=5749)	26.5 (1523)	0.1 (6)	0.5 (30)	0.9 (50)	10.4 (598)	0.2 (10)	5.0 (289)	0 (0)	2.1 (122)	0.1 (4)	1.1 (66)
Cam-pub (n=3982)	5.0 (200)	2.0 (80)	1.7 (66)	0.5 (20)	1.0 (39)	1.3 (50)	0.8 (31)	0.5 (21)	7.0 (277)	0.1 (3)	52.1 (2075)
Ghan-pub (n=7263)	17.6 (1281)	1.7 (120)	0.7 (54)	0.5 (33)	2.9 (213)	2.8 (202)	0.9 (62)	0.01 (1)	7.5 (544)	0.01 (1)	0 (0)
Tanz1-pub^‡^ (n=3456)	8.8 (304)	0.03 (1)	0.1 (4)	0 (0)	17.0 (588)	0.1 (3)	0.5 (17)	0.03 (1)	1.9 (66)	0 (0)	15.9 (548)
Tanz2-pub (n=60 189)	31.5 (18 928)	0.04 (22)	4.8 (2863)	5.4 (3247)	24.5 (14 766)	1.4 (843)	0.8 (499)	0.01 (6)	5.1 (3056)	0 (0)	0.1 (59)
Uga-pub (n=432 513)	18.7 (80 748)	0.02 (100)	0.6 (2586)	1.5 (6421)	32.8 (141 904)	1.6 (6776)	0.01 (45)	1.2 (5339)	6.9 (29 622)	0.1 (448)	0 (0)
Uga-priv^§^ (n=497)	9.3 (46)	0.2 (1)	3.6 (18)	0.4 (2)	9.3 (46)	0.8 (4)	2.2 (11)	0 (0)	4.6 (23)	0 (0)	0 (0)
Nige-mix (n=6588)	2.3 (154)	0.2 (14)	0.4 (23)	0.2 (11)	2.0 (131)	0.7 (48)	0.4 (24)	0 (0)	1.7 (109)	0.02 (10)	10.7 (708)

*Penicillins primarily included oral and injectable penicillin formulations, and amoxicillin with or without clavulanic acid, as well as ampicillin, cloxacillin, dicloxacillin, and flucloxacillin; cephalosporins included first and second generation cephalosporins, as well as ceftriaxone and cefixime; macrolides included azithromycin and erythromycin; tetracycline was typically doxycycline; TMP/SMX=trimethoprim-sulfamethoxazole; quinolones primarily included ciprofloxacin, as well as levofloxacin, nalidixic acid, norfloxacin, and sparfloxacin; metronidazole also included secnidazole, tinidazole; “other” included clindamycin, nitrofurantoin, thalazole, and others.

†Data presented only for Afghanistan study in public health facilities (Afgh-pub/a and Afgh-pub/b). In study among community health workers in Afghanistan (Afgh-com/a and Afgh-com/b) trimethoprim-sulfamethoxazole was only antibiotic available to prescribers.

‡Tanz1 recorded drugs actually obtained by patients; other studies recorded drugs prescribed.

§Only subset of patients (n=497) in Uga-priv were followed up after consultation to collect data on drugs prescribed.

**Figure f3:**
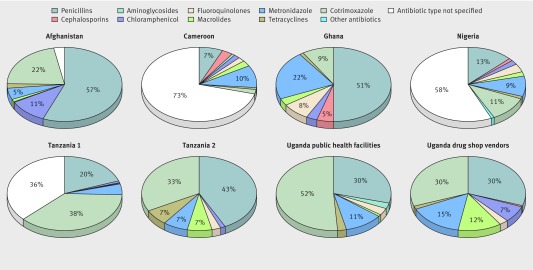
**Fig 3** Antibiotic class as proportion of all antibiotics prescribed in each study (control and intervention settings combined). Afghanistan data are from Afgh-pub only; Afgh-com health workers had access only to trimethoprim-sulfamethoxazole. White areas for Cameroon, Nigeria, Afghanistan, and Tanzania-1 indicate that systemic (oral or injectable) antibiotic was prescribed but that name was not specified in study records. Labels indicate classes that accounted for ≥5% of all antibiotics prescribed for each study

Compared with patients with positive malaria test results, prescription of each of the four most common classes of antibiotic (penicillins, TMP-SMX, tetracyclines, and metronidazole) was higher for patients with negative test results across most sites (table 6). Risk ratios ranged from 1.70 to 28.2 for penicillins, 0.96 to 19.7 for TMP-SMX, 3.21 to 9.0 for tetracyclines, and 1.24 to 3.37 for metronidazole.

**Table 6 tbl6:** Risk ratios (95% confidence interval) for antibacterial prescription by class^*^ for patients with negative *v* test positive test results in rapid diagnostic test for malaria (mRDT) intervention^†^

	Penicillin	TMP/SMX	Metronidazole	Tetracycline
Afgh-pub	28.2 (11.5 to 69)	19.7 (7.2 to 54)	0^‡^	6.5 (0.97 to 43)
Cam-pub	1.70 (1.08 to 2.69)	0.96 (0.47 to 1.94)	1.24 (0.78 to 1.97)	6.3 (0.86 to 46)
Ghan-pub	2.25 (1.72 to 2.95)	3.57 (2.30 to 5.5)	2.64 (1.55 to 4.5)	9.0 (0.94 to 87)
Tanz1-pub^§^	3.21 (1.80 to 5.7)	1.32 (0.88 to 1.98)	0^‡^	0^‡^
Tanz2-pub	3.73 (2.82 to 4.9)	1.87 (1.45 to 2.40)	2.45 (1.38 to 4.3)	3.21 (1.98 to 5.2)
Uga-pub	2.17 (1.68 to 2.80)	1.48 (1.13 to 1.95)	3.37 (2.72 to 4.2)	7.0 (4.7 to 10.5)
Uga-priv	2.00 (0.98 to 4.1)	1.43 (0.70 to 2.91)	2.47 (0.76 to 8.0)	0^‡^

*Penicillins primarily included oral and injectable penicillin formulations, and amoxicillin with or without clavulanic acid, as well as ampicillin, cloxacillin, dicloxacillin, and flucloxacillin; TMP/SMX=trimethoprim-sulfamethoxazole; metronidazole also included secnidazole, tinidazole; tetracycline was typically doxycycline.

†Afgh-com, study among community health workers in Afghanistan, dropped from this analysis because trimethoprim-sulfamethoxazole was only antibiotic to which participating health workers had access. Nige-mix, study in Nigeria, dropped from this analysis as no or few observations in many relevant categories.

‡No or few observations in relevant categories.

§Tanz1 recorded drugs actually obtained by patients; other studies recorded medicines prescribed.

## Discussion

In this analysis of African and Asian studies including over half a million children and adults with acute febrile illness, we found that introduction of rapid diagnostic tests for malaria to reduce unnecessary use of antimalarials—a beneficial public health outcome—could drive up empirical use of antibiotics. Antimicrobial drug resistance can result in prolonged illnesses, higher mortality, and increased costs of treatment and is a major global concern.^7^ Unnecessary overuse of antimicrobials increases drug pressure and contributes to the development and spread of antimicrobial resistance. Acute febrile illness is one of the most common presenting syndromes in tropical and subtropical regions, and patient and prescriber beliefs and behaviours regarding management of fever influence antimicrobial use.^49^ ^50^

Several studies, including the component studies in our analysis, have shown that rapid diagnostic tests for malaria, when combined with effective training, can reduce overuse of antimalarials.^8^ Our current study, however, has shown that the desired reduction in empirical use of antimalarial drugs was often accompanied by an unintended shift toward increased prescription of other antimicrobials, specifically antibiotics. This shift was observed for multiple classes of antibiotics and across several epidemiological and healthcare contexts where rapid diagnostic tests were introduced. In particular, empirical antibiotic use was much more common for patients with negative malaria test results. These findings suggest that without additional interventions, current major initiatives to introduce rapid diagnostic tests for malaria—which could effectively reduce inappropriate use of antimalarials and the risk of antimalarial drug resistance^14^—can unintentionally exchange presumptive overuse of antimalarials for presumptive overuse of antibiotics. The potential for this prescribing shift was recognised in the early days of increasing the use of these tests,^16^ and these concerns now seem to be real in many settings.^18^ There is a widespread assumption that improving pathogen specific diagnosis with better tests will reduce overuse of antimicrobials, but it might simply shift overuse from one class to another.

### Strengths and weaknesses of study

The strengths of the study include the wide range of geographical, epidemiological, and healthcare settings that are typical of contexts where rapid diagnostic tests for malaria will be used and the consistency and size of the effect on antibiotic prescribing. Data from over 520 000 patient encounters in Africa and Asia were available, providing the broadest sample currently available to evaluate shifts in prescribing behaviour associated with test implementation.

As with all studies there are limitations. The ACT Consortium studies were conducted in sites representative of where most patients typically seek treatment. As advanced microbiology diagnostic facilities were not readily available at these sites, the data do not allow determination of whether antibiotic use was appropriate for individual patients. Antibiotic availability varied across study settings, which increases generalisability of the results but also means that sites are not strictly comparable in terms of drugs or classes used. Limitations of individual studies are reported in the published papers on their findings.

This analysis design can robustly identify that there is an increase in antibiotic prescribing after introduction of rapid diagnostic tests for malaria, but it cannot identify the reasons for this shift at an individual prescriber level. Qualitative research would be better suited to answer such questions.

Combination of data from studies with meta-analysis must be undertaken with caution when the data come from highly variable epidemiological settings and different health settings. We therefore consider the summary statistic useful, but it should not be overinterpreted, and the consistency of results across different settings is equally important.

There were no major outbreaks (such as the Ebola epidemic in West Africa) in any of the study sites that might have affected the results.

### Interpretation in light of other studies

It is not possible to know whether antibiotic prescription was appropriate at the individual patient level in this analysis because the studies did not collect full clinical data or samples for further laboratory investigation. In most similar settings where bacterial diagnosis has been undertaken, however, few patients have documented bacterial infections; fewer than 2% (and in virtually all reports <5%) of patients with uncomplicated febrile illness have positive results on blood cultures.^51^ ^52^ ^53^ Not all bacterial causes of fever lead to bacteraemia, but, for example, in young children with uncomplicated febrile illness in Zanzibar, just 22% had an infection retrospectively considered to require antibiotics.^54^ At a population level it is likely that relatively few patients in our studies had bacterial infections requiring antibiotic treatment, and the incidence is unlikely to be anywhere near the 69% suggested by antibiotic prescription to those with negative results of malaria tests.

Case management guidelines for limited resource settings, such as the WHO’s Integrated Management of Childhood Illness (IMCI) and Integrated Management of Adult and Adolescent Illness, do not recommend empirical use of antibiotics for non-severe febrile illness of unclear aetiology.^55^ ^56^ The extent to which these guidelines are used, or adhered to, however, varies by setting. Studies in settings where antibiotics are not available to prescribers, such as chemist shops in Ghana, have shown that withholding both antibiotics and antimalarials diverts patients to antipyretics, which is a safe strategy for most uncomplicated illness.^57^

With declining incidence of malaria in many settings, the proportion of fevers attributable to illnesses other than malaria stands to increase. Currently rapid diagnostic tests are more useful to rule out malaria than to rule it in, at least in Africa, but this is likely to change as the incidence of malaria drops. As this transition occurs and the proportion of negative test results increases, the risk of inappropriate antibiotic treatment of patients with negative results is likely to increase as well. Widespread childhood vaccination for pneumococcus, meningococcus, and *Haemophilus influenzae* type B, and the resulting reduction in bacteraemia and bacterial infection, have further reduced the risk that non-specific, non-severe febrile illness is caused by a potentially serious bacterial pathogen in many African and Asian countries.^58^ ^59^ ^60^

Even when treatment with antibiotics is warranted, patients might not receive the most appropriate antibiotic for their illness, particularly in settings with inadequate healthcare infrastructure.^20^ In the studies we analysed, choice of antibacterial was inevitably untargeted as health workers in these settings do not have access to facilities to confirm diagnoses and identify pathogens nor to epidemiological data to help guide antibiotic choices—a health system weakness that is unfortunately typical across most malaria endemic areas. In the ACT Consortium studies, nearly all antibiotic prescriptions were for penicillins, trimethoprim-sulfamethoxazole, tetracyclines, and, in several sites, metronidazole. The fact that metronidazole prescribing was more common where rapid diagnostic tests for malaria were introduced, similar to the pattern for other antibiotic classes, suggests a relatively haphazard approach to empirical prescribing; few causes of malaria-like febrile illness can be effectively treated with metronidazole.

Within each study site, different antibiotics predominated, which could reflect to varying degrees local availability including stockouts (when healthcare facilities run out) of antibiotics because of intermittent supply,^61^ recommendations in national or other clinical guidelines such as IMCI,^56^ and personal or institutional preferences. Other broad spectrum antibiotics such as cephalosporins, fluoroquinolones, and macrolides were relatively infrequently prescribed in the sites studied and are probably either less available or affordable or are thought to be restricted to particular indications; this could change over the next few years. Of note, at most sites tetracyclines accounted for only a tiny fraction of antibiotics prescribed, even among non-paediatric patients; yet this inexpensive antibiotic class could be a rational empirical choice to cover zoonotic infectious agents such as rickettsiae, leptospira, and several bacteria that cause a considerable proportion of infections in these areas.^17^ ^62^ ^63^ ^64^ Reliable data on antibiotic resistance are scarce to non-existent in regions typified by ACT Consortium study sites^20^; reports that are available from Africa indicate that, for example, currently 1% to 100% of *Streptococcus pneumoniae* isolates are resistance to penicillin, while 0% to 35% isolates of non-typhoidal salmonella are resistant to fluoroquinolones.^65^

Clinical case management typically follows an expected pathway that culminates in the prescription or purchase of medicines.^66^ ^67^ In low resource settings, antimicrobial medicines are often the cornerstone of care.^68^ ^69^ Presentation of fever is expected to result in antimicrobial prescription.^49^ ^70^ ^71^ Behaviour change to reduce unnecessary overuse of antimalarials can occur when introduction of diagnostic technologies is accompanied by a well designed and implemented programme of training and supervision.^37^ ^39^ ^41^

### Meaning of study for policy and clinical practice

This analysis suggests that while introduction of rapid diagnostic tests for malaria can reduce untargeted excessive use of antimalarials—a highly beneficial public health outcome across malaria endemic regions—it can also have the unintended consequence of driving up untargeted and probably excessive use of antibiotics. In this analysis, the shift included antibiotics from several classes and was consistent across nearly all the varied clinical and epidemiological settings studied, increasing the generalisability of the findings. Therefore when rapid diagnostic tests are introduced, policymakers and clinicians should avoid a switch to overuse of antibiotics, a concern that increases the challenges of changing prescribing practice. This awareness is important in the design of programmes for provider training and community education in Africa and Asia, where antibiotic use is already relatively unregulated and unrestricted.^20^ ^24^ ^65^ Without thoughtful intervention in the near term, as the burden of malaria declines and negative malaria test results become more common, the trend toward compensatory prescription of antibiotics can only contribute to increasing levels of antibiotic resistance.

### Unanswered questions and future research

This analysis shows quantitatively that introduction of rapid diagnostic tests for malaria can lead to an increase in antibiotic prescribing in many settings. Two major gaps in evidence need to be filled to inform policy and clinical practice guidelines to deal with this problem. The first is to identify the current drivers of this prescribing behaviour (mainly with qualitative research but also with epidemiology) to inform efforts toward behaviour change. The second, especially important for the rational revision of diagnostic algorithms such as IMCI, is to identify the treatable or preventable causes of non-malaria febrile illness (mainly with microbiology, virology, and epidemiology).

**What is already known on this topic**

Antimalarial drugs are widely overprescribed: introduction of rapid diagnostic tests for malaria reduces overprescription and helps target antimalarial drugs to those who need them
Antibiotics are also widely overprescribed, and antimicrobial resistance poses a fundamental threat to human health, development, and security

**What this study adds**

At the same time as reducing overuse of antimalarial drugs, introduction of rapid diagnostic tests for malaria is associated with markedly increased levels of antibiotic prescribing especially for patients with negative test results
This effect is seen across multiple settings in Africa and Asia and, in large part, probably represents increased overprescribing of antibiotics

## Notes

We gratefully acknowledge the assistance of the clinical staff and patients in all of the studies contributing data for this analysis. We thank Daniel Chandramohan, Bonnie Cundill, Catherine Maiteki, Renata Mandike, Wilfred Mbacham, Richard Ndyomugyenyi, Denise Roth Allen, and Rebecca Thomson for their valuable participation in earlier discussions of the ACT Consortium RDTs in Context working group; Paul Snell for development and management of the central data repository; and Bianca D’Souza, Débora Miranda, Rebecca Tremain, Eleanor Challenger, and Philippe Verstraete for logistical and administrative support for this analysis.

Contributors: KJB, CIRC, EKA, FB, SC, DD, CG, SPK, SL, DGL, TL, PM, LMJ, IM, AKM, OEO, HR, MWR, JW, VLW, SY, DS, SGS, and CJMW contributed to the design and supervision and/or coordination of the nine primary studies on which this analysis is based. All authors participated in the working group that contributed to the idea, design, and interpretation of the analysis. KJB, BL, and HH prepared the data for analysis. KJB analysed the data for the main outcome measures; HH analysed the data for the secondary aim. MEC and BL advised on statistical analysis. HH and KJB had full access to all of the data in the study and take responsibility for the integrity of the data and the accuracy of the data analysis. HH and CJMW drafted the manuscript. All authors critically revised the manuscript for important intellectual content, and read and approved the final paper. HH is guarantor of the paper.

Ethical approval: All studies in this analysis had ethical approval from their host academic institutions and national authorities, as detailed in open access published reports from the individual studies.

Transparency: The lead author affirms that the manuscript is an honest, accurate, and transparent account of the study being reported; that no important aspects of the study have been omitted; and that any discrepancies from the study as planned (and, if relevant, registered) have been explained.

Data sharing: Data from individual studies and a combined dataset used for this analysis are available from the ACT Consortium data repository (https://actc.lshtm.ac.uk/, searchable by author, publication date, or journal) or from the corresponding authors on request. Participant consent for data sharing was not obtained for all studies, but the presented data are anonymised and risk of identification is low.
